# Remarks on the Treatment of Coughs

**Published:** 1906-01

**Authors:** N. Gordon Price

**Affiliations:** Newark, N. J.


### Remarks on the Treatment of Coughs.

By N. GORDON PRICE, M. D., Newark, N. J.

COUGH is a troublesome symptom of a variety of diseases, manisfested by one or more sudden violent expirations, having usually for its object the removal of some irritating substance from the air passages. Cough may be divided into dry and moist, according to the quantity of expectoration; simple and spasmodic, depending on the number of expulsive efforts: laryngeal, tracheal, pleuritic, bronchial etc., according to the seat of the trouble; nervous or sympathetic, resulting from nervous irritation either direct or reflex. The cough attending dentition, disturbances in the nose and pharynx, or an elongated uvula should be placed in the last division.

The correct diagnosis of the underlying condition producing the cough is not a simple matter. Careful cognisance must be taken of every possible source of irritation and inflammation. We are too much in the habit of making such sweeping and comprehensive diagnosis as "cold," "cold on the lungs," etc., without actually satisfying ourselves as to the real seat of the trouble. The pleura, lungs, bronchi, nose, throat, larynx, and uvula, each must be carefully examined, before we can feel assured that we are not merely jumping at conclusions. Frequently, a so called chronic bronchitis, is cured at one sitting by a simple uvulotomy or tonsillotomy.

The treatment of coughs is very frequently ineffectual; particularly is this true of the spasmodic variety accompanying bronchial asthma, pertusis, and subacute and chronic laryngitis. In fact, loss of confidence in the abilities of the physician may be just as frequently traced to his inability to check a cough as to his inability to collect his bills. The converse of this statement is also true; cure a cough and you command confidence. For this reason and also because conditions attended with cough are every day occurrences, a physician desiring to enlarge his practice must be continually on the watch for a remedy which will be reliable and effectual.

We have, it is true, in our pharmacopea several drugs which are recommended for the above conditions; at times they are successful, but in the vast majority of cases they fail us completely. Ipecac, squills, senega, stramonium, etc., if given in full doses to robust individuals tend to alleviate the above conditions by rendering a dry cough moist, but they are exceedingly depressing besides being irritating to the gastrointestinal tract and renal epithelium. For this reason these heroic depleting agencies must be employed in selected cases. Small doses in my experience produce very little influence on the air passages, but do produce a markedly irritating effect on the stomach, manifested by nausea and complete anorexia.

The ideal remedy in bronchial asthma, pertusis, and laryngitis is one which will soothe the congested mucous membrane of the air passages, soften and liquify the tough and tenacious mucus and check spasm; it must furthermore be palatable, non-toxic, non-irritating to the stomach and should be free from the likelihood of imparting the drug habit. These qualities would fairly respond to the nice requirements of up-to-date therapeutics. The attention of the physician is called almost daily to some new mixture or elixir warranted to cure all ailments attended with cough, tuberculosis not excepted, but as a rule they are worse than useless; after a short spasm of popularity, their novelty wears off and sooner or later they find their way to the waste pipes. The great number of these cough mixtures on the market merely indicates that there actually exists a need for a safe and reliable preparation and that the physicians armamentarium in this regard is still incomplete.

About five months ago a medical friend suggested the use of glycoheroin (Smith) in an unsatisfactory case of pertusis, which, on trial, proved quite gratifying. This success coupled with my colleagues high praise induced me to undertake an investigation as to its merits, and though sceptical at first, being adverse to the use of proprietary preparations on general principles, I am now fully convinced that the remedy is destined to supplant the preparations we have formerly placed reliance upon. It possesses all the qualities above outlined as requisites of an ideal expectorant. It produces excellent results in all conditions accompanied by cough, but it has proven particularly successful in the more unyielding cases as, for example, in bronchial asthma, pertusis, and subacute and chronic laryngitis. It is an exact glycerine solution of heroin, combined with expectorant remedies and balsams. Each teaspoonful is equivalent to one-sixteenth grain of heroin; the solution is permanent and unalterable. The adult dose is one teaspoonful given every two or three hours; the dose for children in proportion. In color, taste and odor it meets the requirements of the most fastidious.

A striking feature of this remedial agent is its rapidity of action. Almost unalterably, within a half hour its soothing effect is noticed in allaying an irritable cough. It seems not only to liquify the stringy, tough, mucus clinging to the lining of the air passages, but to all appearances to actually force it into the vault of the pharynx, so that the intense strain incident to the efforts at expulsion are practically done away with. Besides, it soothes and so to speak lulls into quiescence, the perturbed nervous system, inducing refreshing slumber,—an effect most desirable in these conditions; it also sharpens the appetite and generally speaking invigorates the patient.

The only unfavorable influence which I have occasionally observed is that it is prone to render the bowels slightly constipated, in those with a sluggish circulation. Any aperient salt, however, at once overcomes this tendency. I have also convinced myself that raw fruit taken at breakfast or a glass of hot water imbibed on rising, obviates the necessity of taking any laxative on the part of these patients.

In bronchial asthma, glycoheroin works almost like a charm. Within a short time it causes the expectoration of a large quantity of ball-like masses, eases the harassing cough, allays the dyspnea and thus bridges over the attack in a pleasant and rapid manner. The continued use of this remedy in the intervals of the attacks diminishes their frequency and severity. I can safely state that no other remedy has produced such brilliant and effectual results in this annoying ailment, at least as far as my individual experience goes.

Whooping cough is still, so to speak, an untamed mustang, running wild; we are still without the magic bridle with which to tether it. Perhaps in the future its exact etiology will be unriddled; perhaps a special preventive and curative antitoxin will be discovered. But at the present time we can merely dream of a specific in this condition. Belladonna and the expectorants have from time immemorial been prescribed in whoping cough and physicians as a rule, irrespective of the negative results, dare not swerve in their allegiance to this custom. When we have prescribed belladonna and obtain no result we inform the parents of the little ones that nature will cure the condition, or else advise an extended sojourn at the sea shore, ostensibly to enable the little ones to inhale the odoriferous and health-imbued ozone of the atmosphere, but actually to rid ourselves of troublesome patients. Glycoheroin has produced excellent results in a limited number of cases of pertusis and I would suggest a fair and impartial trial of this remedy by the medical rofession at large. I do not wish it understood that I recommend it as a specific or antidote, but I do wish to state that it shortens the duration of the disease, diminishes the severity of the paroxysms of coughing and for this reason minimizes the likelihood of complications.

In subacute and chronic laryngitis I have obtained good results with glycoheroin. As would be expected in tubercular laryngitis the preparation has produced very little influence so far as the course of the disease is concerned; it has however added considerably to the comfort of the sufferers. To illustrate the action of glycoheroin I desire to append a few of the numerous cases in which I have employed this remedy. I shall cite only cases of bronchial asthma, whooping cough, and subacute and chronic laryngitis, because these conditions have heretofore baffled all my efforts and caused me the loss of the confidence of many a patient.

Case I.—Was hurriedly summoned to attend Mrs. H. B., a lady of 45, poorly nourished, very nervous and excitable. She was in the throes of an attack of bronchial asthma, had been troubled with asthma for nine years. Her breathing was gasping and laborious, accompanied by a wheezing sound which could be heard all through the house. She coughed frequently and in spasms, alarming in severity. Ordered teaspoonful doses of glycoheroin to be given hourly until the subsidence of the attack. She took three doses and the attack subsided; she expectorated large balls of phlegm, her breathing became almost normal, the wheezing disappeared, and she was able to partake of a hearty meal. Soon afterwards she fell into a deep sleep, and on waking felt much refreshed. Continued glycoheroin in teaspoonful doses four times a day for four weeks. Patient was delighted with the new remedy; she informed me that she had never before passed her "asthma season" with so little discomfort. In addition to this remedy I resorted to tonics and feeding to improve her debilitated condition.

Case II.—D. M., a policeman 38 years of age, rather over fed, afflicted with chronic bronchitis, asthma and emphysema, called me because of his asthmatic attacks which were frequent and severe:—at the time of my first visit he was in the agonies of an attack. Ordered, iodides with stramonium and squills: could not tolerate the mixture, vomited incessantly and became enfeebled; ten hours afterward no subsidence of the attack. Then I ordered glycoheroin in teaspoonful doses every hour. Signs of improvement within three hours; dyspnea and spasmodic cough allayed, wheezing disappeared and patient shortly afterwards fell into a refreshing sleep. Continued glycoheroin four times a day. The attacks were diminished both in frequency, severity and duration, his gastric irritability subdued and general condition improved. Two weeks after beginning of treatment, patient declared himself entirely relieved.

Case III.—Four children in the same family ranging from 1 to 8 years, afflicted with whooping cough; the two youngest had the disease in a more violent form than the two oldest. I deemed it an excellent opportunity to test the actual value of glycoheroin in pertusis. For the two oldest, ordered a mixture containing belladonna, stramonium, and squills, and the two youngest glycoheroin. To my surprise the two youngest began to show positive signs of improvement, whereas the condition of the two oldest seemed to grow worse; their paroxysms were more frequent and severe and their general tone became lowered. After a weeks' experimentation, became convinced that glycoheroin was far superior to the belladonna mixture. I therefore placed the two oldest also on this remedy. The four children made a pleasant and uneventful recovery within four weeks after commencement of treatment.

Case IV.—Girl of 5 developed whooping cough after an attack of measles. The paroxysms of coughing were extremely distressing; her eyes were inflamed and edematous, her tongue fissured and inclined to bleed. She had been treated with negative results by a colleague who had been dosing her with antipyrine. Gave her ten drop doses of glycoheroin every two hours and insisted on having her in the open air the greater part of the day and gave the mother general directions concerning the proper ventilation of sleeping apartments. The child began to improve the first day after treatment began. In less than four weeks the disease was entirely eradicated. I should have mentioned the fact that the antipyrine had produced a cyanosis in the child of over six hours duration, much to the horror and fright of the parents.

Case V.—R. E., a vaudeville singer, age 28, robust, athletic, handsome. Complained of hoarseness and inability to continue at his vocation because of continual tickling and change in tone of his voice. Feared he would completely lose his voice. Had already consulted a specialist of note but received no benefit from his medicines. I thought it a good opportunity to test glycoheroin. Much to my surprise and satisfaction in a few days he reported improvement. Continued the remedy and in two weeks he was able to use his voice again, all hoarseness had disappeared; he continued the remedy for two weeks longer in smaller doses in fear of a relapse.

Case VI.—J. F., a cantor, 46 years of age, short and broad, with good chest expansion, consulted me with hoarseness that had prevented him from continuing his vocation for five months. Gave him expectorants, local treatment, but without avail. Finally I resorted to glycoheroin with remarkable results. He rapidly improved, and in three weeks was able to use his voice fairly well. In four weeks he was perfectly well. As far as I know he has had no recurrence.

In conclusion I desire my colleagues to give this admirable remedy a fair test in appropriate cases, and I feel confident that it will elicit their hearty approval.

62 Boston Street.

